# A comparative study of large-vessel and small-vessel primary angiitis of the central nervous system: insights from a Chinese single-center retrospective cohort

**DOI:** 10.3389/fimmu.2025.1724588

**Published:** 2025-12-17

**Authors:** Yifan Wang, Jiangyi Lyu, Fangfang Li, Jing Zhao, Yu He, Shangtian Xiang, Weizhou Zang

**Affiliations:** 1 Department of Neurology,Zhengzhou University People’s Hospital, Zhengzhou, China; 2 Department of Neurology, Henan Provincial People’s Hospital, Zhengzhou, China; 3 Department of Neurology, Henan University People’s Hospital, Zhengzhou, China

**Keywords:** primary central nervous system vasculitis, clinical features, imaging features, prognosis, CNS immune diseases

## Abstract

**Background:**

Primary angiitis of the central nervous system (PACNS) is a rare immune-mediated vasculitis with distinct subtypes (large/medium-vessel [LV-PACNS] and small-vessel [SV-PACNS]). This study aims to provide evidence-based data from the Chinese population for improving the subtype-specific management system of PACNS.

**Methods:**

This retrospective single-center study enrolled 47 PACNS patients (29 with SV-PACNS, 18 with LV-PACNS) who met the 1988 Calabrese and Mallek diagnostic criteria, with a follow-up duration ≥1 year. Clinical phenotypes, imaging characteristics, treatment, prognosis, and factors influencing prognosis were retrospectively analyzed in both groups.

**Results:**

Compared with LV-PACNS, SV-PACNS had more severe initial neurological impairment (baseline modified Rankin Scale mRS score: 3 vs. 2, p=0.043) and a longer median time from onset to treatment initiation (154 days vs. 58.5 days, p=0.013). In terms of clinical features, LV-PACNS had a higher incidence of cerebrovascular events (88.9% vs. 58.2%, p=0.027) and limb weakness/sensory abnormalities (83.3% vs. 34.5%, p=0.001), while SV-PACNS had a higher incidence of tumor-like lesions (41.4% vs. 5.6%, p=0.008). Imaging features showed that 100% of LV-PACNS patients had cerebrovascular stenosis, of which 94.4% exhibited circumferential vascular wall enhancement; and compared with SV-PACNS, they had a higher incidence of ischemic infarction (66.7% vs 20.7%, p=0.002). Multivariate analysis confirmed that time from onset to treatment was an independent risk factor for poor 1-year prognosis (mRS score > 2) in both subtypes (SV-PACNS: OR = 1.012, p=0.021; LV-PACNS: OR = 1.048, p=0.040).

**Conclusions:**

This study identified the core clinical and neuroimaging differences between LV-PACNS and SV-PACNS, and pointed out that treatment delay are the main issues affecting prognosis.

## Introduction

Primary angiitis of the central nervous system (PACNS) is an immune-mediated inflammatory disease that is limited to the vascular walls of the brain and spinal cord (1), primarily affecting the arteries within the brain parenchyma, spinal cord, and soft meninges, with rare involvement of veins. According to research statistics, the incidence rate of PACNS is approximately 2.4 per 1,000,000 (2). PACNS can occur at any age, with a male-to-female ratio of approximately 0.8:1 (2). PACNS can affect blood vessels of various sizes within the cranium. Large-vessel primary angiitis of the central nervous system (LV-PACNS) is mainly characterized by involvement of large/medium-sized vessels(clinically, the proximal segments and secondary branches of cerebral arteries are usually classified as large/medium-sized vessels (3)). Its common neurological manifestations are nonspecific, with the most frequent clinical presentations in order being limb weakness, cognitive impairment, focal neurological symptoms, headache, and visual disturbance (4). Small-vessel primary angiitis of the central nervous system (SV-PACNS) affects smaller blood vessels (assessable only by biopsy). Its common clinical manifestations are similar to those of LV-PACNS but also have some differences. The most common clinical presentations of SV-PACNS in order are cognitive impairment, epileptiform seizures, focal neurological symptoms, headache, visual disturbance, etc (4).

Magnetic resonance imaging (MRI) is essential for diagnosing PACNS, but it is also nonspecific. Possible imaging findings (5) include multifocal or bilateral infarcts, intracerebral hemorrhage, subarachnoid hemorrhage, and multiple microbleeds, as well as gadolinium enhancement of the parenchyma and leptomeninges, and even tumor-like lesions. Regarding differences between large-vessel and small-vessel subtypes, recent studies have shown that diffuse cortical microhemorrhages accompanied by atrophy (the “coral sign”) may be an important MRI pattern suggestive of SV-PACNS (6). In contrast, LV-PACNS is more likely to present with acute, subacute, or chronic infarcts in the distribution areas of large blood vessels, as well as abnormal vascular wall changes—such as concentric thickening of the vascular wall, concentric enhancement of Grade I (enhancement intensity lower than the pituitary stalk) to Grade II (enhancement intensity equal to or higher than the pituitary stalk), and negative remodeling—detectable by high-resolution magnetic resonance vessel wall imaging (HR-VWI) (7). Following treatment with hormones or immunosuppressive agents, vascular wall enhancement may partially or completely disappear. This dynamic change is a key criterion for distinguishing vascular inflammatory (8–10).Computed tomography (CT) also plays a certain role in the diagnosis of PACNS, but its sensitivity is lower than that of MRI. Cerebral angiography is valuable for diagnosing LV-PACNS,commonly used examinations include digital subtraction angiography (DSA), magnetic resonance angiography (MRA), and computed tomography angiography (CTA). The European Stroke Organization guidelines define high-probability vasculitis angiographic patterns as (2): segmental alternating stenosis and dilation of cerebral arteries (“beaded sign”), involving multiple vessels with arterial occlusion, in the absence of proximal atherosclerosis or other recognized vascular abnormalities. However, due to the limitation of cerebrovascular examinations in visualizing blood vessels with a diameter of less than 5 mm, cerebrovascular imaging studies in patients with SV-PACNS often yield no positive findings (11), and a normal angiographic result cannot completely rule out the diagnosis of SV-PACNS.

Approximately 80–90% of PACNS cases exhibit abnormal cerebrospinal fluid (CSF) findings (12), but these changes are nonspecific. Common CSF abnormalities include mild elevations in white blood cell count, protein content, or both. In terms of differences between large-vessel and small-vessel subtypes, current studies have not identified CSF markers with clear discriminatory significance.

Numerous international cohort studies have confirmed that patients with LV-PACNS and SV-PACNS exhibit distinct differences in clinical manifestations and imaging features (4, 13–16). However, subtype-specific comparative studies focusing on the Chinese population remain relatively scarce. Current guidelines have not yet established stratified management strategies based on vessel size classification, and differences in treatment responses and long-term outcomes between the two subtypes still require further validation with more population-specific data. Treating patients with different vessel involvement patterns as a homogeneous group may hinder a comprehensive understanding of the heterogeneity of the PACNS clinical spectrum. Therefore, this study aims to supplementarily analyze the clinical features, treatment responses, and prognostic patterns of LV-PACNS and SV-PACNS based on a Chinese single-center cohort, providing evidence-based data from the Chinese population to improve the subtype-specific management system for PACNS.

## Materials and methods

### Patients

**Figure 1:**
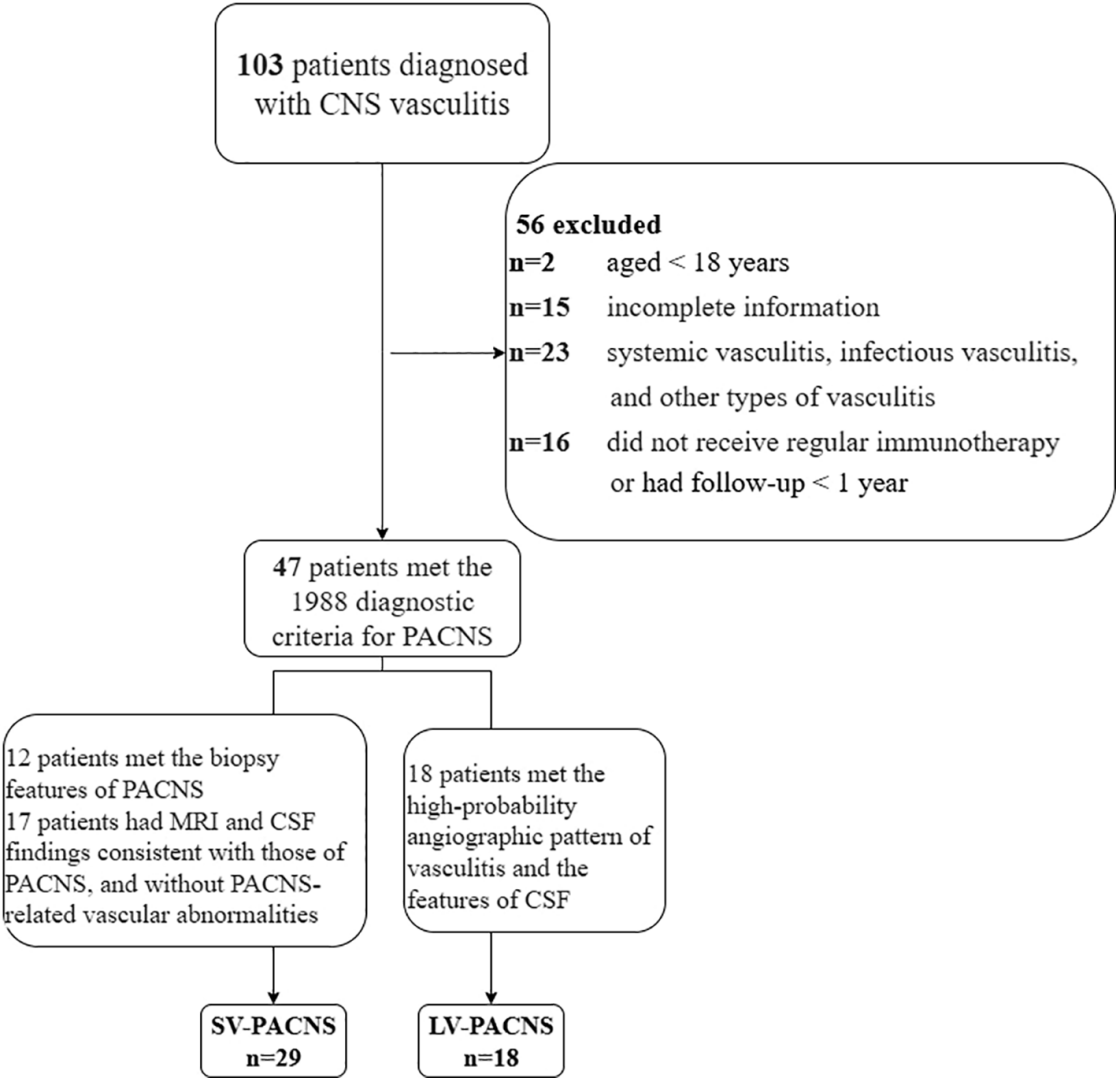
Flowchart of patient screening and enrollment in this single-center retrospective study.

From January 2017 to December 2024, Henan Provincial People’s Hospital diagnosed a total of 103 cases of CNS vasculitis. After review and follow-up, we ultimately included 47 cases of PACNS patients who met the inclusion criteria for this study. The inclusion criteria are as follows: (1) Patients met the preliminary diagnostic criteria for PACNS established by Calabrese and Mallek in 1988 (17): acquired neurological deficits that cannot be explained by other diseases, imaging or biopsy features consistent with vasculitis, no evidence of systemic or secondary vasculitis; and the diagnosis was made after discussion by two experienced neurologists (W.Z. and F.L.); (2) Patients received regular treatment and were followed up for at least one year (or died within one year); (3) Patients were aged ≥18 years. Patients were classified into the LV-PACNS group and the SV-PACNS group based on cerebral vascular examination and brain biopsy results. LV-PACNS was defined as patients with biopsy features of vasculitis or lacking biopsy data, meeting the high-probability angiographic pattern and CSF features of vasculitis; SV-PACNS is defined as patients with biopsy features of vasculitis or lacking biopsy data, with MRI and CSF findings consistent with PACNS, and no vascular abnormalities observed in cerebral vascular examinations. The patient screening and selection process in our study is shown in **Figure 1**.

### Data collection

Data were collected from electronic medical records, covering: (1) Baseline demographics such as sex, age at symptom onset, age when treatment started, and existing comorbidities like hypertension, hyperlipidemia, diabetes, previous or active infections (hepatitis B/C, HIV, syphilis), rheumatic or immune-mediated diseases, and cancer history; (2) Clinical signs including motor and sensory deficits, visual problems, aphasia or dysarthria, headache, seizures, cognitive issues, fever, dizziness or vertigo, myelopathy, psychiatric or behavioral changes, and ataxia; (3) Laboratory tests including serology (ESR, CRP, homocysteine, autoimmune and rheumatic panels, antiphospholipid antibodies, lupus anticoagulant) and CSF analysis (opening pressure, cell count, biochemistry, oligoclonal bands, viral PCR to rule out other diagnoses), with the rates of abnormal results calculated for ESR, CRP, CSF proteins, and CSF leukocytosis; (4) Neuroimaging and histopathology: MRI (with and without contrast), HR-VWI, angiography (DSA, CTA, MRA), and brain biopsy findings; (5) Treatment details involving initial therapy and ongoing immunosuppressive treatment; (6) All patients’ neurological function was assessed via the electronic medical record system before treatment and at 1 year after treatment (with an extended follow-up to 2 years for some patients). Additionally, telephone follow-up was conducted to monitor the current status of each patient.

### Biopsy

The definitive diagnosis of PACNS currently relies on brain tissue biopsy. However, due to the limitations of brain biopsy, the sensitivity of PACNS biopsy ranges from 53% to 63% (18). Brain tissue biopsies were conducted by the Department of Neurosurgery at Henan Provincial People’s Hospital, utilizing stereotactic brain biopsy (SBB) or lesion resection guided by frame-based multimodal imaging. SBB combines imaging data—such as contrast-enhanced CT, contrast-enhanced T1- and T2-weighted MRI scans, and MRA—into the Elekta SurgeryPlan system for image fusion, allowing for targeted sampling of abnormalities. The system plans the biopsy path, calculates the Leksell frame coordinates for each axis, and determines the scalp biopsy points based on these coordinates for precise stereotactic procedures. The ideal biopsy sample should contain the cortex, soft meninges, dura mater, and superficial white matter (12).

### Clinical evaluation of MRI examinations and MR images

MRI examinations were conducted using a 3.0T Siemens MAGNETOM scanner equipped with a 64-channel head/neck coil. All patients underwent standardized sequences: conventional brain MRI (T1WI, T2WI, T2-FLAIR, DWI) and 3D-TOF-MRA. Selected patients received an additional 3D-HR-VWI with dark-blood technique (C4 to circle of Willis coverage). Imaging evaluations by blinded neuroradiologists included: (1) ischemic injury (DWI/ADC for acute, T2WI/FLAIR for chronic infarction); (2) hemorrhagic manifestations (parenchymal hemorrhage, microbleeds, SAH via T2*/SWI); (3) LV-PACNS vascular pathology (DSA-confirmed stenosis, post-contrast HR-VWI wall enhancement); (4) inflammatory features (parenchymal/leptomeningeal enhancement); (5) tumefactive inflammatory foci.

### Scale assessment and prognosis definition

In this study, we used the modified Rankin Scale (mRS) to quantify the neurological function of the two groups of patients before treatment (baseline), one year after treatment, and in some patients, extended to two years after treatment. A baseline mRS score >2 was defined as severe initial neurological damage; a follow-up mRS score ≤2 was defined as good prognosis. For mRS dynamic changes: Clinical improvement: A decrease in follow-up mRS score by ≥1 point from baseline. Disease relapse: Specifically refers to the recurrence of new symptoms related to the pathological mechanism of the original disease, or a significant exacerbation of existing symptoms, in neurological diseases after a period of “remission”. Relapse criteria for this study: ① The new symptoms/exacerbation of symptoms lasts for ≥24 hours; ② The interval from the previous remission period is ≥30 days; ③ Confirmed by diagnostic methods, such as the presence of new lesions shown by brain magnetic resonance imaging (MRI), and an increase of ≥1 point in the modified Rankin Scale (mRS) score compared with that during the remission period. Disease progression: Refers to the persistent and irreversible deterioration of the condition that occurs during the course of neurological diseases. It does not require the prerequisite of “having achieved remission previously” and can occur during treatment or follow-up of the natural disease course. Progression criteria for this study: ① Progressive exacerbation of core symptoms; ② Imaging or biomarkers indicating an expansion of the lesion range or an increase in the pathological severity; ③ The modified Rankin Scale (mRS) score increased by ≥ 1 point compared with the baseline score.

### Statistical analysis

Statistical analysis was performed using SPSS 22.0 statistical software. Continuous variables that followed a normal distribution were expressed as mean ± standard deviation, and comparisons between two means were performed using the t-test; continuous variables that did not follow a normal distribution were expressed as median (interquartile range), and comparisons between two groups were performed using the Mann-Whitney U test; categorical variables were expressed as frequency and percentage, and comparisons between groups were performed using the chi-square test and Fisher’s exact probability test. In this study, two-tailed tests were used for the comparative analysis of clinical baseline characteristics between the two groups. *P* < 0.05 was considered statistically significant, while no multiple comparison correction was performed.

The mRS score was treated as a multiclass ordered variable. Clinical outcomes (improvement, no improvement, relapse/worsening/death) and good outcomes of SV-PACNS and LV-PACNS groups were evaluated at baseline, 1-year and 2-year follow-up; ΔmRS (1-year post-treatment minus baseline mRS) was analyzed too, with hypothesis testing (*p* < 0.05) for intergroup differences. Kaplan-Meier analysis plotted recurrence-free survival curves, and log-rank test compared the curves between groups.

Univariate and multivariate logistic regression analyses were conducted to identify risk factors associated with adverse prognosis, where the patient’s prognostic status was defined as the dependent variable (favorable prognosis = 0, adverse prognosis = 1) and the following independent variables were included: continuous variables including age, neutrophil-to-lymphocyte ratio (NLR), CSF protein-to-serum protein ratio (Qalb), treatment delay time, and baseline mRS score, and dichotomous categorical variables including gender (female = 0, male = 1), high-risk factors (hypertension, diabetes mellitus, dyslipidemia; all coded as absent = 0, present = 1), clinical symptoms and signs (headache, limb weakness, epilepsy; all coded as absent = 0, present = 1), imaging features (intracranial hemorrhage, leptomeningeal enhancement, mass effect, multiple infarctions; all coded as absent = 0, present = 1), and treatment regimen (hormone monotherapy = 0, hormone combined with immunosuppressants = 1), with variables with *p* < 0.1 in the univariate analysis included in the multivariate logistic regression model and variables with *p* < 0.05 in the multivariate analysis identified as independent prognostic factors.

## Result

### Patient demographics

A total of 47 patients were included, with 20 (42.6%) being female. Among these, 29 cases were in the SV-PACNS group and 18 cases were in the LV-PACNS group. Fifteen patients (31.9%) underwent brain biopsy, with 13 cases in the SV-PACNS group, of which 12 (92.3%) were positive (6 cases of lymphocytic vasculitis, 4 cases of granulomatous vasculitis, and 2 cases of necrotizing vasculitis), while both cases in the LV-PACNS group were negative.

A comparison of clinical data between the SV-PACNS and LV-PACNS groups showed that the mean age at onset was slightly higher in the SV-PACNS group than in the LV-PACNS group (48 years vs. 42.5 years, *p* = 0.347), but the difference was not statistically significant. The median time from first onset to diagnosis in the SV-PACNS group was 2.7 times that of the LV-PACNS group (153 days vs. 57 days, p=0.014). The median time from first onset to initiation of treatment in the SV-PACNS group was 2.6 times that of the LV-PACNS group (154 days vs. 58.5 days, p=0.013). All patients underwent serological and cerebrospinal fluid examinations to rule out other causes. Serological abnormalities included elevated erythrocyte sedimentation rate (ESR>20 mm/h), elevated CRP (CRP > 10 mg/L), and elevated NLR, while CSF abnormalities included elevated white blood cell count (WBC > 8 × 10^6^/L), elevated protein levels (Pro > 0.45 g/L), and elevated CSF-serum protein ratio (Qalb) (Albs, serum albumin; Albcsf, CSF albumin; Qalb, ratio of Albcsf to Albs) (**Figure 1**). There were no statistically significant differences in serum abnormalities or cerebrospinal fluid abnormalities between the two groups (*p* > 0.05).

**Figure 2:**
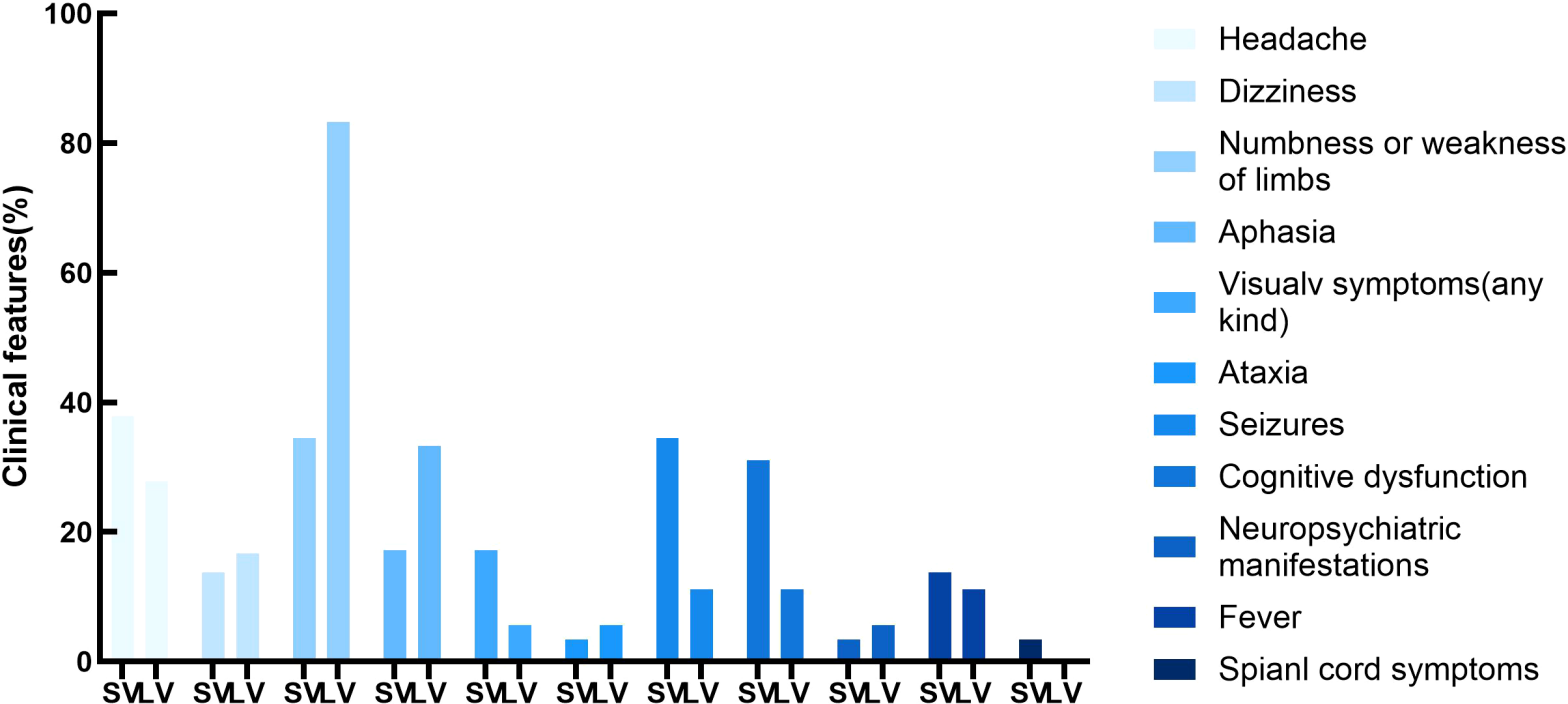
Spectrum of clinical manifestations in PACNS.

In the SV-PACNS group, 17 patients (58.2%) had focal neurological deficits (including limb weakness or sensory abnormalities, speech disorders, visual symptoms, and ataxia), 16 patients (55.2%) had encephalopathy (seizures, cognitive impairment, and psychiatric and behavioral abnormalities), 11 patients (37.9%) experienced headaches, 4 patients (13.8%) experienced dizziness or vertigo, 4 patients (13.8%) experienced fever, and 1 patient (3.4%) experienced symptoms of spinal cord involvement (thoracic and lumbar spine). Patients in the LV-PACNS group had a higher incidence of cerebrovascular events compared to the SV-PACNS group (88.9% vs. 58.2%, *p* = 0.027), particularly limb weakness/sensory abnormalities (83.3% vs. 34.5%, *p* = 0.001), while encephalopathy was less common(55.2% vs. 27.8%, p=0.066), with no statistical significance. In the LV-PACNS group, 5 patients (27.8%) experienced headaches, and 2 patients (11.1%) experienced fever (**Figure 2**).

**Figure 3:**
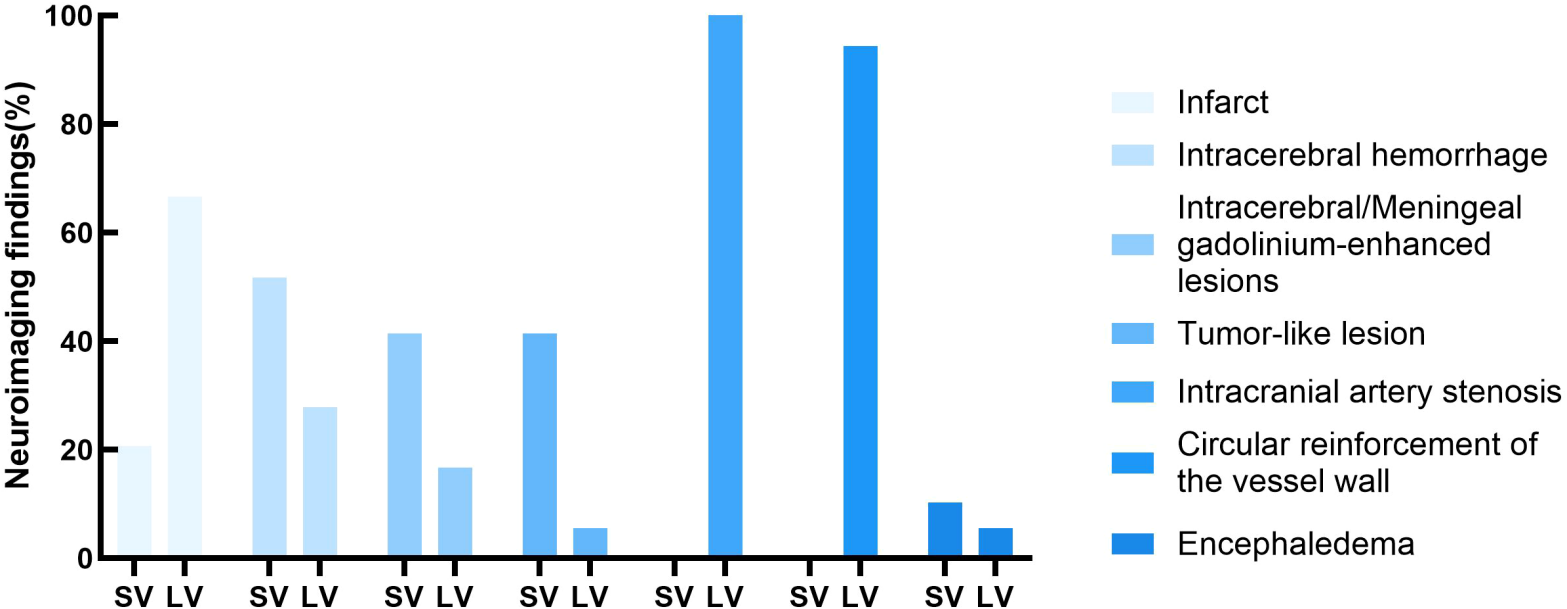
Distribution of major neuroimaging abnormalities in PACNS.

Ischemic infarction was more common in the LV-PACNS group than in the SV-PACNS group (66.7% vs. 20.7%, *p* = 0.002).Tumor-like lesions were more common in the SV-PACNS group than in the LV-PACNS group (41.4% vs. 5.6%, *p* = 0.008). Compared to the LV-PACNS group, the SV-PACNS group had a higher incidence of intracranial hemorrhage (51.7% vs. 27.8%, p=0.107) and intracerebral parenchymal or pia mater enhancement (41.4% vs. 16.7%, p=0.077), with no statistical significance for either. All patients in the LV-PACNS group underwent DSA examination, and all patients had intracranial arterial stenosis. All LV-PACNS patients underwent HR-VWI examination, and 17 cases (94.4%) showed circumferential enhancement of the vascular wall (**Figure 3**).

The 47 patients enrolled in the study all received first-line immunotherapy during the acute phase (1 g/day methylprednisolone injection as an initial dose, administered for 3 consecutive days, followed by gradual tapering; or combined with 1g cyclophosphamide administered once a month). Nine patients in the SV-PACNS group and four patients in the LV-PACNS group upgraded their treatment regimen after first-line therapy failed or multiple relapses occurred, receiving rituximab (1 g per dose, with the second dose administered 14 days after the first, totaling two doses, repeated every 6–9 months). There was no statistically significant difference in acute-phase treatment regimens between the SV-PACNS and LV-PACNS groups (p > 0.05). All patients receiving immunotherapy continued maintenance therapy with low-dose steroids or mycophenolate mofetil for at least 12 months (**Table 1**).

**Table 1:** Comparison of clinical features and treatment between SV-PACNS and LV-PACNS patients.

Baseline characteristics		SV-PACNS (n=29)	LV-PACNS (n=18)	*p*
Gender, n (%)	Female	10 (34.5)	10 (55.6)	0.155
Age at onset, years		48 (25)	42.5 (18)	0.319
Onset-to-diagnosis time, M (IQR)		153 (194)	57 (103.25)	0.014
Onset-to-treatment time, M (IQR)		154 (225.5)	58.5 (106.75)	0.013
Underlying conditions, n (%)		9 (31.0)	6 (33.3)	0.869
Inflammatory markers	Elevated ESR, n (%)	10/28 (35.7)	6/16 (37.5)	0.906
	Elevated CRP, n (%)	6/20 (30.0)	11/18 (61.1)	0.054
	NLR, M (IQR)	2.38 (1.63)	1.82 (1.46)	0.096
Cerebrospinal fluid parameters	Elevated CSF white blood cell count,n (%)	22 (75.9)	9 (50)	0.069
	Elevated CSF protein, n (%)	22 (75.9)	12 (66.7)	0.521
	CSF-serum protein ratio Qalb, M (IQR)	14 (5)	14 (6)	0.886
Biopsy, n (%)	Number of biopsies	13 (44.8)	2 (11.1)	0.016
	Positive biopsy	12 (92.3)	0 (0)	0.029
Clinical manifestations, n (%)	Headache	11 (37.9)	5 (27.8)	0.475
	Dizziness	4 (13.8)	3 (16.7)	1.000
	Focal neurological deficits	17 (58.2)	16 (88.9)	0.027
	Numbness or weakness of limbs	10 (34.5)	15 (83.3)	0.001
	Aphasia	5 (17.2)	6 (33.3%)	0.291
	Visual symptoms (any kind)	5 (17.2)	1 (5.6)	0.384
	Ataxia	1 (3.4)	1 (5.6)	1.000
	Encephalopathy	16 (55.2)	5 (27.8)	0.066
	Seizures	10 (34.5)	2 (11.1%)	0.095
	Cognitive dysfunction	9 (31.0)	2 (11.1)	0.164
	Neuropsychiatric manifestations	1 (3.4)	1 (5.6)	1.000
	Fever	4 (13.8)	2 (11.1)	1.000
	Spinal cord symptoms	1 (3.4)	0 (0)	1.000
Imaging features, n (%)	Infact	6 (20.7)	12 (66.7)	0.002
	Intracerebral Hemorrhage	15 (51.7)	5 (27.8)	0.107
	Intracerebral/Meningeal gadolinium-enhanced Lesions	12 (41.4)	3 (16.7)	0.077
	Tumor-like lesion	12 (41.4)	1 (5.6)	0.008
	Intracranial artery stenosis (DSA/CTA/MRA)	0 (0)	18 (100)	-
	Circular reinforcement of the vessel wall	0/4 (0)	17 (94.4)	-
	Encephaledema	3 (10.3)	1 (5.6)	1.000
Treatment, n (%)	Glucocorticoid monotherapy	8 (27.6)	6 (33.3)	0.675
	GC + Cyclophosphamide	21 (72.4)	12 (66.7)	0.675
	With adjunctive rituximab	9 (31.0)	4 (22.2)	0.739

“-” indicates no data/no statistical need; ESR, erythrocyte sedimentation rate; CRP, C-reactive protein; NLR, Neutrophil-to-lymphocyte ratio; Seizures, at least one unprovoked epileptic seizure caused by abnormal neuronal discharge; DSA/CTA/MRA, angiographic examinations; GC, glucocorticoid.

### Outcome

Further statistical analysis treated the modified Rankin Scale (mRS) as a multi-class ordered variable. After one year of treatment, 20 patients (69.0%) in the SV-PACNS group achieved clinical improvement, 5 patients (17.2%) showed no significant improvement, and 4 patients (13.8%) experienced relapse or worsening. In the LV-PACNS group, after one year of treatment, 10 patients (55.6%) achieved clinical improvement, 3 patients (16.7%) showed no significant improvement, and 5 patients (27.8%) experienced relapse or worsening. Following one year of immunotherapy, the SV-PACNS group demonstrated a higher rate of clinical improvement compared to the LV-PACNS group; however, this difference was not statistically significant (69.0% vs. 55.6%; *p* = 0.352).

**Figure 4:**
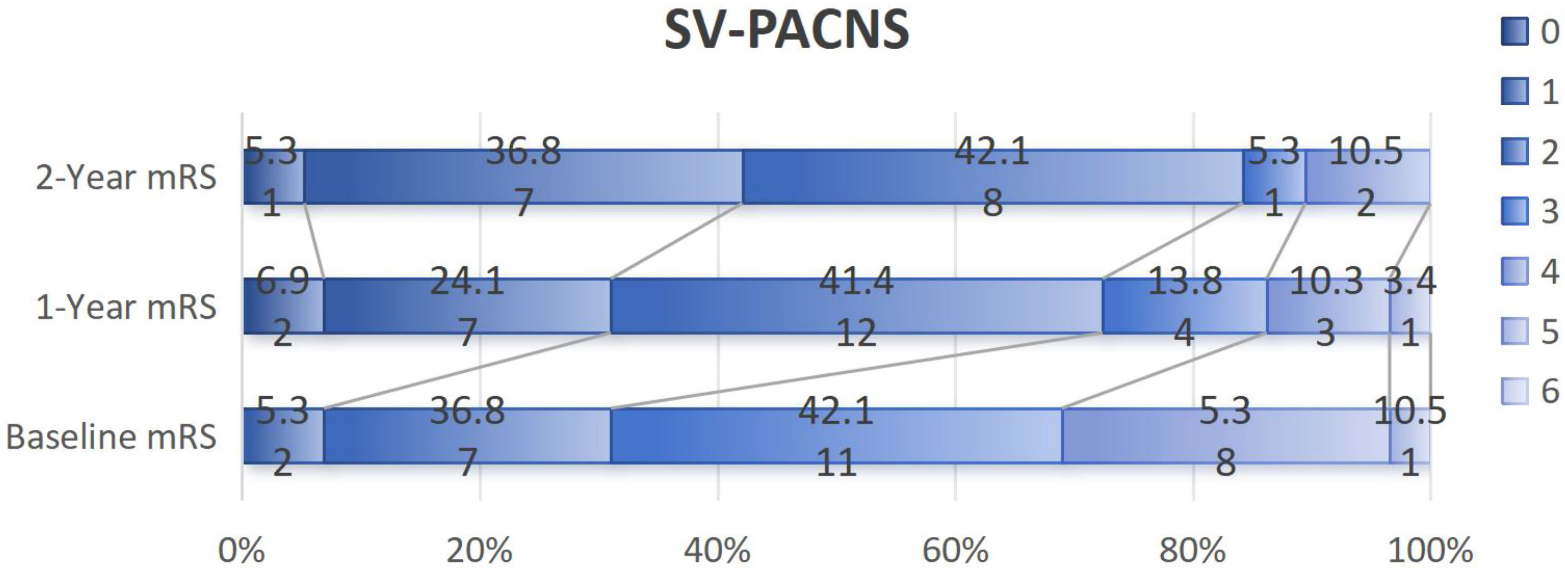
mRS Trajectory in SV-PACNS: baseline to 2-year follow-up.

**Figure 5:**
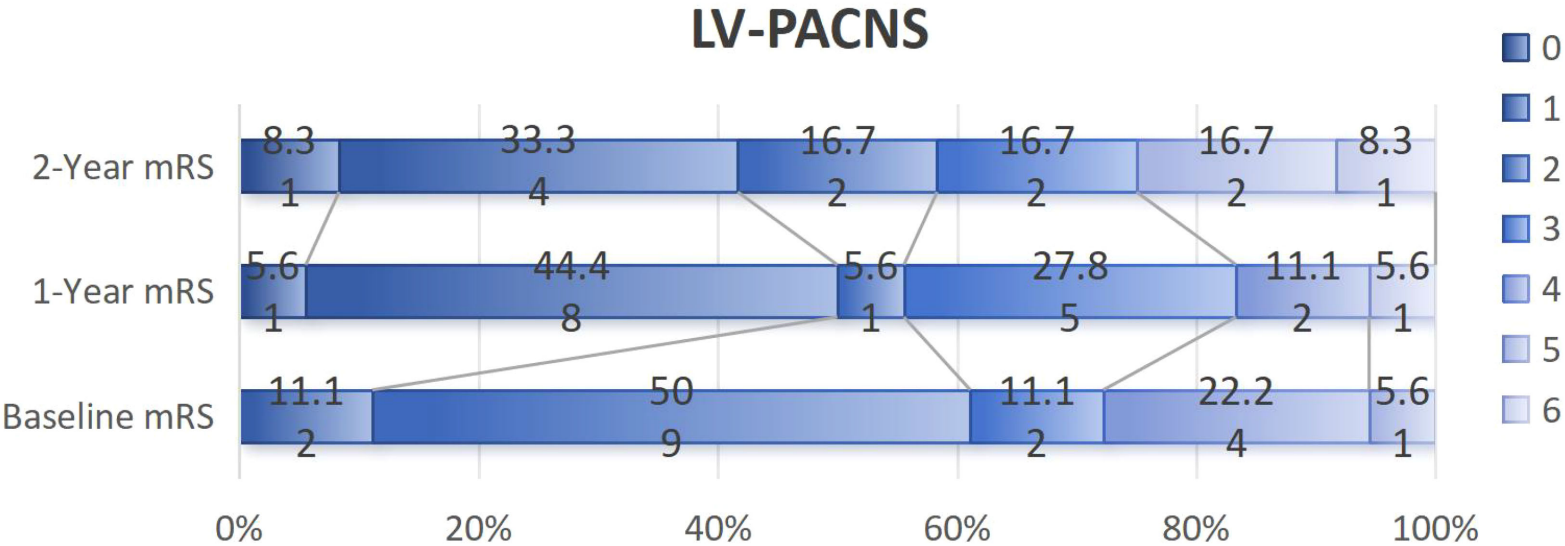
mRS trajectory in LV-PACNS: baseline to 2-year follow-up.

Patients in the SV-PACNS group had more severe initial neurological damage than those in the LV-PACNS group (3 points vs. 2 points, *p* = 0.043). After one year of treatment, 21 out of 29 patients (72.4%) in the SV-PACNS group had good outcomes, while 10 out of 18 patients (55.6%) in the LV-PACNS group had good outcomes; After 2 years of treatment, 16/19 (84.2%) patients in the SV-PACNS group had good outcomes, while 7/12 (58.3%) patients in the LV-PACNS group had good outcomes, but the difference was not statistically significant (**Tables 2**, **3**, **Figures 4**, **5**).

**Table 2:** Comparison of functional outcomes by mRS in SV-PACNS vs. LV-PACNS subtypes.

Time	Group	Total	mRS		p
			≤2	>2	
Baseline	SV-PACNS	29	9	20	0.043
	LV-PACNS	18	11	7	
1 year	SV-PACNS	29	21	8	0.236
	LV-PACNS	18	10	8	
2 year	SV-PACNS	19	16	3	0.206
	LV-PACNS	12	7	5	

**Table 3:** Change in mRS scores at 1-year follow-up in SV-PACNS vs. LV-PACNS patients.

ΔmRS	≤-1 Improvement	0 No improvement	≥1 Relapse/worsening/death
SV-PACNS	20	5	4
LV-PACNS	10	3	5
*p*	0.352	1.000	0.274

ΔmRS, mRS score after 1 year of treatment - baseline mRS score.

Finally, we used the Kaplan-Meier survival analysis method to plot the recurrence-free survival curves for the SV-PACNS group and the LV-PACNS group, and compared the differences in recurrence-free survival between the two groups using the log-rank test. The results showed that the recurrence-free survival curves of the SV-PACNS group and the LV-PACNS group had similar downward trends, and there was no statistically significant difference in recurrence-free survival rates between the two groups (*p* = 0.528).

### Multivariate analysis of prognostic factors in PACNS

**Figure 6:**
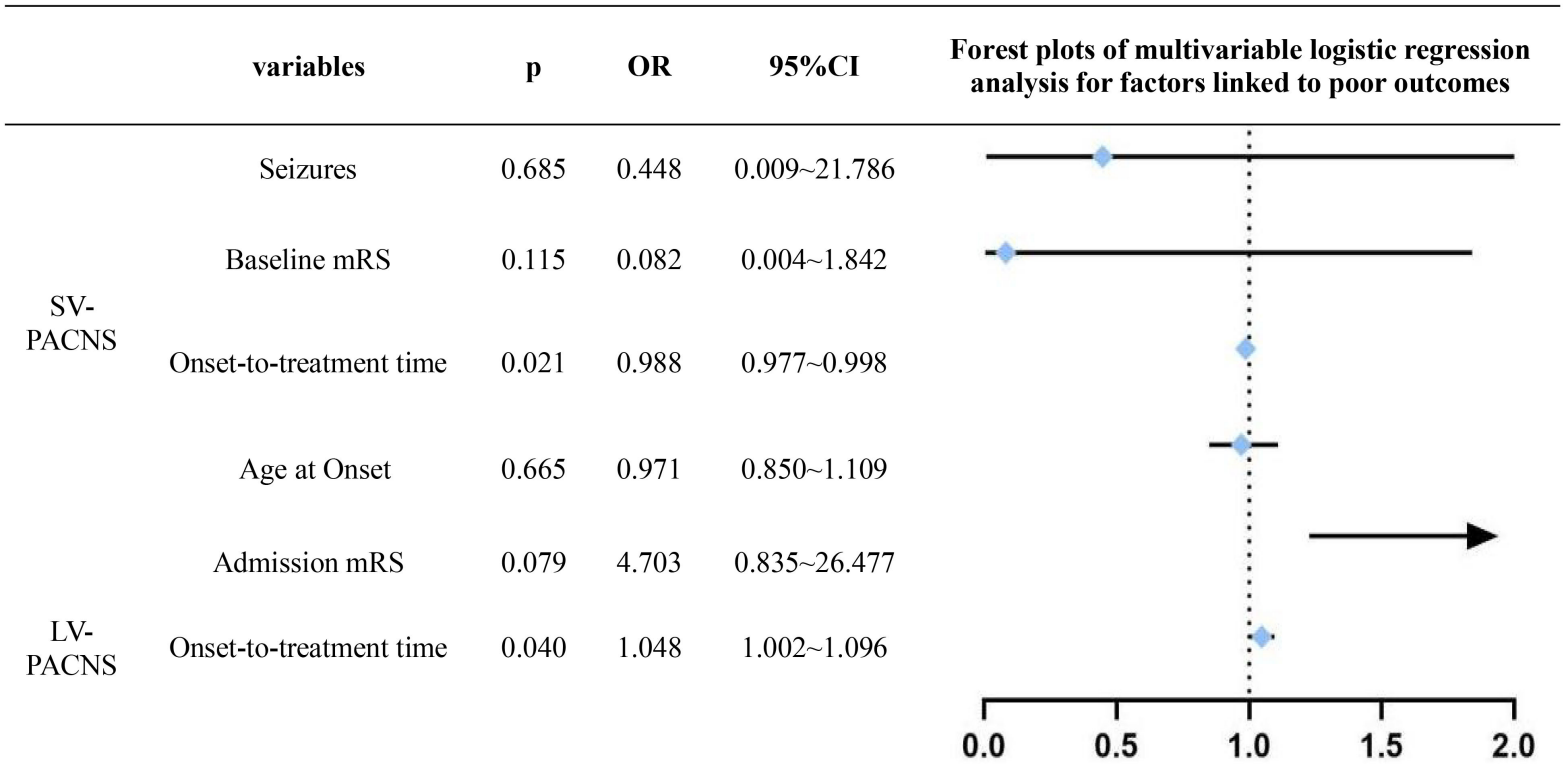
Multivariate logistic regression analysis of factors associated with poor prognosis.

Univariate logistic regression analysis revealed that age at onset (p=0.076), time from onset to diagnosis (p=0.039), time from onset to treatment (p=0.041), baseline mRS (p=0.039), and seizures (p=0.061) were associated with 1-year outcomes in the SV-PACNS group. In the LV-PACNS group, time from onset to treatment (p=0.088) and baseline mRS (p=0.052) were correlated with 1-year outcomes. Multiple linear regression analysis was performed to explore the impact of the aforementioned factors on patient prognosis. Multicollinearity was assessed using variance inflation factor (VIF) and tolerance tests. The results showed that the VIF values of time from onset to diagnosis and time from onset to treatment were 386.993 and 383.842, respectively (both > 10), with tolerance values of 0.003 each (both < 0.1), indicating severe multicollinearity between the two variables. Considering that time from onset to treatment directly reflects the interval from disease onset to the initiation of intervention and is more clinically relevant for intervention, only time from onset to treatment was included as the core time-related independent variable in the final model, while other variables (age at onset, baseline mRS score, and seizures history) were retained for analysis. In the optimized model, the VIF values of all variables were ≤ 2.025, indicating that the multicollinearity issue had been effectively controlled. Multivariate logistic regression analysis of the aforementioned factors demonstrated that time from onset to treatment was a common independent risk factor for 1-year outcomes in both SV-PACNS and LV-PACNS patients (SV-PACNS: p=0.021, OR = 1.012, 95%CI 1.002–1.023; LV-PACNS: p=0.040, OR = 1.048, 95%CI 1.002–1.096) (**Figure 6**).

## Discussion

This study systematically analyzed the clinical features, outcomes, and disease course of two major subtypes of PACNS —SV-PACNS and LV-PACNS subtypes. PACNS is a rare yet devastating intracranial vasculitis; its marked clinical heterogeneity frequently leads to delayed diagnosis and suboptimal treatment. Using a single-center cohort with detailed follow-up, we aimed to identify key discriminative features of these subtypes, screen prognosis-related modifiable factors, and offer evidence to optimize clinical decision-making.

There were no significant differences in gender, age at onset, serological inflammatory markers, cerebrospinal fluid (CSF) white blood cell count, or CSF protein content between the two patient groups. The median time from first onset to diagnosis in the SV-PACNS group was 2.7 times that in the LV-PACNS group (p=0.014), and the median time from first onset to treatment was 2.6 times longer in the SV-PACNS group compared with the LV-PACNS group (p=0.013). These results suggest that the diagnosis and treatment of SV-PACNS pose greater challenges than those of LV-PACNS, which may be attributed to the non-specificity of non-invasive examinations and the excessive reliance on brain biopsy for SV-PACNS diagnosis. As an invasive procedure, brain biopsy has limitations including low patient acceptance, low sensitivity (50% to 75% (12)), and strict requirements for sampling inherent to the technique itself. Therefore, although brain biopsy is the gold standard for PACNS diagnosis, emphasizing the differences in clinical manifestations and neuroimaging features between SV-PACNS and LV-PACNS and implementing early intervention and treatment are also of paramount importance. In our cohort, the brain biopsy results of 2 cases in the LV-PACNS group were negative. Based on previous research findings (biopsies of PACNS may be due to irregular involvement of intracranial vessels or negative sampling of normal brain regions (19), the difference between biopsy-positive and negative PACNS that may reflect the size of the involved vessels (20)), and by integrating the patients’ clinical manifestations, CSF results, and imaging examinations, we consider the results to be false negatives, which may be related to the inability to sample large blood vessels and the absence of involvement of small blood vessels.

In terms of clinical manifestations, more pronounced neurological deficits were exhibited in the LV-PACNS group. In contrast, improvement or even complete resolution of limb weakness/numbness was often observed in the SV-PACNS group following immunotherapy. Conversely, stroke-like events were frequently experienced by the LV-PACNS group due to large vessel stenosis/occlusion, resulting in more persistent focal neurological deficits (such as hemiplegia, aphasia, and visual symptoms). In the SV-PACNS group, which is characterized by extensive microvascular involvement, neurological manifestations such as epileptic seizures, cognitive impairment, or functional deficits associated with white matter lesions were more commonly present, consistent with the findings of a systematic review (21). Unlike previous studies, headache was documented as the sole presenting symptom in only a small number of patients; it was more commonly observed as an accompanying symptom during the disease course.

Imaging studies hold significant diagnostic value for PACNS. Intracranial arterial stenosis was observed in all LV-PACNS patients, and the incidence of ischemic infarction was significantly higher compared to the SV-PACNS group. High-resolution vessel wall imaging (HR-VWI) revealed circumferential enhancement and centripetal wall thickening in 95.5% of LV-PACNS patients, suggesting that chronic vascular wall inflammation, chronic inflammatory proliferation, or fibrosis represent the core pathogenic mechanisms in PACNS (22). In contrast, ischemic infarcts in SV-PACNS primarily manifested as multiple cortical and deep small infarct lesions (23), with the underlying pathophysiological mechanism potentially involving inflammation-related microembolism or hypoperfusion (24). Studies have demonstrated that SV-PACNS can present with a characteristic “coral-like sign” on 7-Tesla MRI—characterized by diffuse cortical microbleeds accompanied by cortical atrophy—which is closely associated with inflammatory small vessel involvement (6). This characteristic sign was also detected in eight SV-PACNS patients within the present cohort, further confirming its diagnostic specificity for SV-PACNS. The detection rate of the “coral-like sign” in the present cohort (8/29 cases, 27.6%) was lower than previously reported. This discrepancy is attributable to two primary factors. First, limitations in equipment resolution were observed: 3T MRI demonstrates insufficient sensitivity for detecting gyral microbleeds, hindering clear identification of such subtle lesions, whereas 7T MRI, with superior spatial resolution and sensitivity, significantly improves detection efficiency. Second, this radiological sign requires the co-occurrence of two defining features—diffuse cortical microbleeds and cortical atrophy. Several patients exhibited only isolated manifestations, which did not fulfill the diagnostic criteria and were consequently excluded from statistical analysis. In SV-PACNS, inflammatory involvement of small vessels results in vascular wall disruption due to inflammatory exudation, predisposing to microbleeds or parenchymal hemorrhage. Conversely, the lower hemorrhage risk observed in the LV-PACNS group may be associated with reduced perfusion pressure secondary to collateral circulation development following large vessel stenosis (25). Therefore, vigilance for hemorrhagic transformation risk is warranted in SV-PACNS patients receiving immunosuppressive therapy. Serial monitoring of microbleed burden using dynamic susceptibility-weighted imaging (SWI) sequences is recommended to inform therapeutic decision-making. The incidence of parenchymal/pial enhancement and tumor-like lesions was significantly higher in the SV-PACNS group compared to the LV-PACNS group, consistent with multiple prior studies (14, 16, 26, 27) and supporting the hypothesis proposed by Salvarani et al. that “small vessel inflammation preferentially involves the pia mater” (11). Such lesions typically demonstrate uniform nodular enhancement with mild perilesional edema on MRI, potentially mimicking glioma or lymphoma. This imaging pattern suggests underlying pathological processes in SV-PACNS, including increased vascular fragility, blood-brain barrier disruption, and inflammatory infiltration (28). In summary, the distinct clinical and neuroimaging profiles observed between LV-PACNS and SV-PACNS cohorts reflect divergent underlying vascular pathophysiological mechanisms. LV-PACNS is predominantly characterized by steno-occlusive vasculopathy leading to ischemic injury, whereas SV-PACNS is associated with destructive microvascular pathology.

In this cohort, corticosteroid-cyclophosphamide combination therapy was predominantly administered during the acute phase to both groups, while treatment escalation to rituximab was implemented in refractory cases or patients experiencing multiple relapses. The initial neurological impairment in the SV-PACNS group was significantly more severe than that in the LV-PACNS group (baseline mRS score: 3 vs. 2, p=0.043). However, the rates of favorable prognosis (mRS ≤ 2) at 1 and 2 years after treatment both showed a more favorable trend in the SV-PACNS group (1-year: 72.4% vs. 55.6%; 2-year: 84.2% vs. 58.3%), the intergroup differences did not reach statistical significance (1-year p=0.236; 2-year p=0.206), which may be related to the small sample size of this cohort (only 12 patients in the LV-PACNS group completed the 2-year follow-up). This finding is consistent with the conclusion from the Mayo Clinic series that the small-vessel vasculitis subtype portends a better prognosis across racial groups (26). However, this result is significantly different from the report by Paramasivan et al. from India (13). The results of this Indian cohort showed that the long-term prognosis of SV-PACNS patients was worse than that of the LV-PACNS group (proportion of mRS > 2 at the last follow-up: 72% vs. 34%, p=0.005). The core reason for this discrepancy may be the “treatment timing window” effect caused by the difference in the time interval from symptom onset to the start of treatment. In the Indian cohort, the treatment delay of the SV-PACNS group was as long as 620.5 days, which was much longer than the 118 days of the LV-PACNS group (p=0.001). Excessively long delay may lead to the extensive formation of irreversible neurological damage, thereby limiting the efficacy of immunosuppressive therapy. In our cohort, although the initiation of treatment in the SV-PACNS group was also later than that in the LV-PACNS group (154 days vs. 58.5 days, p=0.013), the delay duration may still be before the neurological function damage was completely solidified. Due to the timely and effective intervention, the final prognosis was favorable. In addition, potential population differences in genetic or environmental factors may affect the distribution of disease phenotypes. Therefore, actively shortening the treatment cycle is the key to improving the prognosis of SV-PACNS. Long-term follow-up data from this study indicate a comparable long-term recurrence risk between the SV-PACNS and LV-PACNS groups. This finding suggests that despite differential vascular involvement between the two PACNS subtypes, their long-term recurrence patterns may share similarities.

Further analysis of prognostic factors revealed that in the univariate model, age, time from onset to treatment, time from onset to diagnosis, baseline mRS, and seizures were significantly associated with 1-year outcomes. Multivariate analysis further confirmed that time from onset to treatment was an independent risk factor affecting the 1-year outcomes of patients in both groups. As an immune-mediated vasculitis, PACNS is characterized by persistent inflammatory damage to blood vessel walls. Therefore, accelerating the diagnostic pathway and initiating intervention and treatment as early as possible are the top priority for improving prognosis. The mortality rate in this study was 4.3%, significantly lower than the rates reported in other cohorts (8%–28%) (13, 16, 29–31). This discrepancy may be attributable to the relatively shorter follow-up duration in our study as well as differences in the characteristics of the study populations. Specifically, this study represents a single-center experience, whereas the comparison cohorts likely included patients from referral centers or multicenter studies with a higher proportion of refractory cases and potentially more severe disease presentations. For more relevant information on cohort comparisons, see **Supplementary Table 1**, which summarizes the key data of a total of 6 cohorts, including those from Germany, the United States (Cleveland Clinic, Mayo Clinic), France, India, and the cohort of this study, and provides a cross-country comparative reference for clinical research on PACNS. In summary, this study demonstrates that treatment delay is a key modifiable factor influencing PACNS prognosis, with particular clinical significance for the LV-PACNS subtype. Future investigations could expand sample sizes, incorporate specific biomarker profiling, and systematically characterize the disease progression patterns of distinct PACNS subtypes to more precisely identify factors associated with prognosis.

### Limitations

This study was a retrospective research. The biopsy rate of patients in the SV-PACNS group was only 44.8% (13/29). As the gold standard for PACNS diagnosis, pathological biopsy plays a crucial role; however, this low biopsy rate led to a considerable proportion of cases relying on a comprehensive diagnosis based on clinical symptoms, imaging findings, and laboratory tests. This may introduce diagnostic bias, thereby overestimating or underestimating the association between imaging features and SV-PACNS. Additionally, key data including clinical manifestations, imaging characteristics, and functional outcomes were all extracted through medical record review, making it inevitable to encounter issues such as recall bias and incomplete documentation.

Despite these limitations, this study reduced confounding from other neurological disorders via strict inclusion criteria, included typical clinicoradiological cases and complete follow-up data, ensuring result validity and evidence for PACNS disease course research. Future plans include extended follow-up and large-sample, multicenter prospective studies. Standardizing biopsies, diagnostic criteria, and treatment will further reduce bias, providing higher-level evidence for PACNS precision medicine and promoting clinical standardization.

## Conclusion

This study identified the core clinical and neuroimaging differences between LV-PACNS and SV-PACNS, highlighting that diagnostic delay and treatment delay are key barriers to favorable prognosis. Clinically, LV-PACNS is defined by ischemic infarcts and limb weakness/sensory abnormalities, while SV-PACNS presents with non-stroke features: tumor-like lesions and severe baseline neurological impairment. Treatment delay is the primary barrier to favorable outcomes. Clinicians need to clearly distinguish the characteristics of the two subtypes, shorten diagnostic delay, and improve the prognosis of PACNS patients through early intervention. In the future, it is necessary to optimize non-invasive diagnostic tools and establish exclusive screening protocols for different PACNS subtypes.

## Data Availability

The original contributions presented in the study are included in the article/**Supplementary Material**. Further inquiries can be directed to the corresponding author.

## Supplementary material

Supplementary Table 1 Comparative Table of International Multicenter Cohorts for PACNS. SVV, small vessel vasculitis; LMVV, large/middle vessel vasculitis; HR-VWI, high-resolution vessel wall imaging; DSA, digital subtraction angiography; GC, glucocorticoid; CYC, cyclophosphamide; IV, intravenous.
