# Supplementary material for: A comparative study of large-vessel and small-vessel primary angiitis of the central nervous system: insights from a Chinese single-center retrospective cohort
Source: Front Immunol. 2025 Dec 17;16:1724588. doi: 10.3389/fimmu.2025.1724588 (PMC12753934; doi:10.3389/fimmu.2025.1724588)
Supplement: Supplementary Table 1 — Comparative Table of International Multicenter Cohorts for PACNS. SVV, small vessel vasculitis; LMVV, large/middle vessel vasculitis; HR-VWI, high-resolution vessel wall imaging; DSA, digital subtraction angiography; GC, glucocorticoid; CYC, cyclophosphamide; IV, intravenous. [file Table1.pdf]

Supplementary Table 1: Comparative Table of International Multicenter Cohorts for PACNS

**Comparison Dimension**	**German Cohort (n=163)[1]**	**Cleveland Clinic Cohort, USA (n=34)[2]**	**Mayo Clinic Cohort, USA (n=191)[3]**	**French Cohort (n=112)[4]**	**Indian Cohort (n=72)[5]**	**Henan Provincial People's Hospital Cohort, China (n=47)**
I. Basic Cohort Information						
Enrolling Institutions	13 tertiary neurological hospitals in Germany (e.g., St. Josef Hospital, Ruhr University Bochum; University Hospital Muenster)	Neurovascular Center and Department of Rheumatic and Immunologic Disease, Cleveland Clinic, USA	Department of Neurology, Rheumatology, and Pathology, Mayo Clinic, USA	Over 20 hospitals in France (French Vasculitis Study Group, French Neurovascular Society, etc.)	Department of Neurology and Imaging Sciences, Sree Chitra Tirunal Institute for Medical Sciences and Technology, Trivandrum, India	Department of Neurology, Henan Provincial People's Hospital, China
Enrollment Period	January 2004 – December 2018	March 2012 – December 2019	January 1983 – December 2017	Retrospective enrollment before 2010; Prospective enrollment 2010–2017	January 2000 – December 2019	January 2017 – December 2024
Subtype Definition	Biopsy-confirmed：Confirmed by biopsy + positive results from any type of cerebrovascular , examinationAngiogram-Confirmed：Positive results from any type of cerebrovascular examination	SVV : Vasculitis in smaller distal branches or normal angiography,<br>LMVV: Vasculitis in proximal or middle arterial segments	Biopsy-confirmed:Angiography-negative+biopsy-positive,<br>Angiogram-confirmed:Angiography-positive	SVV: Biopsy-positive, angiography-negative;<br>LMVV: angiography-positive	SV-PACNS:biopsy-positive, angiography-negative.<br>LV-PACNS:angiography-positive (biopsy-negative or not performed)	SV-PACNS:biopsy-positive,MRI-positive,angiography-negative;<br>LV-PACNS :biopsy-positive,MRI-positive,angiography-positive;

Comparison Dimension	German Cohort (n=163)[1]	Cleveland Clinic Cohort, USA (n=34)[2]	Mayo Clinic Cohort, USA (n=191)[3]	French Cohort (n=112)[4]	Indian Cohort (n=72)[5]	Henan Provincial People's Hospital Cohort, China (n=47)
Sample Size(Subtype Breakdown)	Biopsy-confirmed: 105/163(64.4) (predominantly SV), Angiogram-confirmed: 58/163(35.6) (predominantly LV)	SVV: 23/34 (67.6), LMVV: 11/34(32.4)	Biopsy-confirmed: 71/191(37.2), Angiogram-confirmed: 120/191(62.8)	SVV: :27/112 (24), LMVV: 85/112 (76)	SV-PACNS:18/72 (25), LV-PACNS: 50/72(75)	SV-PACNS: 29 (61.7), LV-PACNS: 18 (38.3)
Main Diagnostic Methods :Positived/Total Tested (Positive Rate %)	Biopsy: 105/120 (77), Angiography: 52/101 (51)	HR-VWI: 12/24(50) SVV VWE positive: 2/14(14.3), LMVV VWE positive: 10/10(100). Brain biopsy: 22/25 (88), SVV: 18/18 (100), LMVV: 4/7 (57.1).	Biopsy: 71/96(74), Cerebral angiography: 129/149 (87), MRI: 95/176 (54).	Biopsy: 33/112 (29), DSA: 68/112 (61), MRA: 11/112 (10), MRI (gadolinium enhancement): 42/92 (46)	DSA: 54/70 (77.1), Brain biopsy: 22/44 (50), HR-VWI: 20/21 (95.2)	LV-PACNS: DSA/CTA/MRA:18/18 (100), HR-VWI: 17/18 (94.4), SV-PACNS: Brain biopsy: 12/13(92.3), MRI+CSF:29(100)
Median Follow-up Duration	42 months	16 months	19 months	57 months	692 days≈22.7 months	≥12 months
II. Baseline Characteristics						

Comparison Dimension	German Cohort (n=163)[1]	Cleveland Clinic Cohort, USA (n=34)[2]	Mayo Clinic Cohort, USA (n=191)[3]	French Cohort (n=112)[4]	Indian Cohort (n=72)[5]	Henan Provincial People's Hospital Cohort, China (n=47)
Median age at diagnosis, yr (range, IQR)	Biopsy-confirmed: 49 (15–83, IQR: 39–63), Angiogram-confirmed: 48 (20–85, IQR: 39.5–58)	SVV: 48 (36–57, IQR: -), LMVV: 47 (35–53, IQR: -)	Biopsy-confirmed: 58 (17–84, IQR: -), Angiogram-confirmed: 48 (17–85, IQR: -)	ALL:47 (range: 18–81, IQR: -)	SV-PACNS:30.5 (24–39, IQR:-), LV-PACNS:40.5 (34–49, IQR: -)	SV-PACNS: 48 (19–59, IQR: 57.5-32.5), LV-PACNS: 42.5 (28–59, IQR: 32..5-50.5)
Female, n (%)	Biopsy-confirmed: 50/105 (48); Angiogram-confirmed: 23/58 (40)	SVV: 7/23 (30.4); LMVV: 6/11 (54.5)	Biopsy-confirmed: 30/71(42.3), Angiogram-confirmed: 72/120(60.0)	ALL:52(46)	SV-PACNS:5/18 (28), LV-PACNS:14/50 (28)	SV-PACNS: 10/29 (34.5), LV-PACNS: 10/18 (55.6)
Median time from primary manifestation to diagnosis, m (IQR)	Biopsy-confirmed: 3 (IQR: 1–19), Angiogram-confirmed: 2 (IQR: 1–5)	SVV: 15.6 (IQR: 4.8–43.2), LMVV: 25.2 (IQR: 2.4–4.4)	Biopsy-confirmed: 2.4 (IQR: 0.2–10.1), Angiogram-confirmed: 1.2 (IQR: 0.1–5.2)	-	Time from first visit to hospital to diagnosis: SV-PACNS: 4.25 (IQR: 0.6–12.0), LV-PACNS: 0.38 (IQR: 1.2–17)	SV-PACNS:5.1 (IQR: 6.5) LV-PACNS: 1.9 (,IQR: 3.4)
Core Clinical Manifestations	Biopsy-confirmed: seizures (47.6%), microhemorrhages (18.4%), mass lesions (35.0%), Angiogram-confirmed: hemiparesis (54.4%), acute infarcts (67.2%), hypertension	SVV:meningeal/parenchymal contrast enhancement (87%), tumor-like lesions (34.8%), seizures (34.8%), LMVV: brain infarcts (100%), strong/concentric vessel wall	Biopsy-confirmed: cognitive dysfunction (66.2%), gadolinium-enhanced lesions (72.7%), Angiogram-confirmed: persistent neurologic deficit/stroke (55.8%),	SVV: headache (51%), cognitive decline (40%), seizures (33%); LMVVl: stroke-like episodes (62%), focal weakness (58%), hypertension (35%)	SV-PACNS: headache (33%), cognitive decline (11%), seizures (22%), abnormal CSF (88%);LV-PACNS: stroke (62%), hemiparesis (70.8%), dysarthria (55.6%), abnormal	SV-PACNS: Encephalopathic manifestations (55.2%), intracranial hemorrhage (51.7%), mass-like lesions (41.4%), gadolinium enhancement (41.4%); LV-PACNS: Focal neurological

Comparison Dimension	German Cohort (n=163)[1]	Cleveland Clinic Cohort, USA (n=34)[2]	Mayo Clinic Cohort, USA (n=191)[3]	French Cohort (n=112)[4]	Indian Cohort (n=72)[5]	Henan Provincial People's Hospital Cohort, China (n=47)
	(50.0%), smoking (46.6%)	enhancement (90%)"	infarcts on MRI (68.2%), hypertension (37.8%)		HR-VWI (90%)	deficits (88.9%), ischemic infarcts (66.7%)
III. Treatment Regimens						
Induction Therapy (%)	Steroid monotherapy: 73/163(44.8), Steroid + CYC: 36/163(22.1)	GC±CYC34/34(100)	Prednisone alone 72/191(37.7); Prednisone + CYC 90/191(47.1,); Prednisone + other immunosuppressants 29/191(15.2); IV methylprednisolone pulses: 86/191(45.5)	GC alone:20/112(17.9), GC + CYC:89/112(79.5), GC + Rituximab: 3/112(2.6), IV methylprednisolone pulses: 68/112(61)	GC alone 45/72(62.5), GC + CYC 27/72(37.5), IV methylprednisolone pulses: 42/72(58)	Methylprednisolone±CYC 47(100), Refractory cases: Rituximab: (SV-PACNS: 9/29(31.0); LV-PACNS: 4/18(22.2))
Maintenance Therapy (%)	Proportion not specified, Drugs: azathioprine, mycophenolate mofetil (immunosuppressive agents)	Proportion not specified, Drugs: azathioprine, mycophenolate mofetil, Duration: median 18 months	19% (35/191) of patients received maintenance therapy. Drugs: azathioprine19/35 (54.3), mycophenolate mofetil8/35 (22.9),methotrexate 5/35(14.3); Median duration: 17 months	Prescribed in 46%(52/112) of patients; Drugs: azathioprine 41/52(79), methotrexate 7/52(14), mycophenolate mofetil 4/52(8), Median duration: 24 months	Prescribed in 98.6% (71/72) of patients, Corticosteroids,:38/71 (52.8), Azathioprine/mycophenolate mofetil/rituximab:33/71(45.8), Median duration: 15 months	Prescribed in 100% of patients, Drugs: low-dose steroids or mycophenolate mofetil, Duration: ≥12 months
IV. Key Outcomes						
Remission	- (Reduced relapse in	LMVV: 10/11(90.9%),	All: 84% (148/177);	All: 106/112 (95);	50/72(69.4) at 6 months，	SV-PACNS: 21/29(72.4)at 1 year,

Comparison Dimension	German Cohort (n=163)[1]	Cleveland Clinic Cohort, USA (n=34)[2]	Mayo Clinic Cohort, USA (n=191)[3]	French Cohort (n=112)[4]	Indian Cohort (n=72)[5]	Henan Provincial People's Hospital Cohort, China (n=47)
Rate (%)	+CYC-treated groups)	SVV: 22/23(95.7%)	Prednisone alone: 83% (58/70);<br>Prednisone + CYC: 81% (69/85);<br>Prednisone + other immunosuppressants: 95% (18/19)	Group 1 (GC alone):13/14(93),<br>Group 2 (GC+immunosuppressant no maintenance): 38/40(95),<br>Group 3 (GC+immunosuppressant + maintenance): 45/45(100)	37/53(69.8%) at 1 year	16/19/(84.2)at 2 years,<br>LV-PACNS: 10/18(55.6) at 1 year,<br>7/12(58.3) at 2 years
Relapse Rate (%)	Biopsy-confirmed: 48/105 （46%）;<br>Angiogram-confirmed: 34/58 （59%）	SVV: 5/23(21.7%);<br>LMVV:7/12(58.3%)	All: 30% (58/191);<br>1 relapse: 33/58,<br>2 relapses:14/58,<br>≥3 relapses:11/58	All:36/106(34),<br>Group 1: 4/14(29),<br>Group 2: 20/40(50),<br>Group 3: 9/45(20)	All: 35/72(48.6),<br>SV-PACNS:16/18(89),<br>LMVV:15/50(30)	All:9/47(19.1),<br>SV-PACNS:4/29(13.8),<br>LV-PACNS: 5/18(27.8)
Poor Outcome Rate (%)	All: (52)	SVV: 8/23(34.8%);<br>LMVV: 6/11(54.5%)	All: 61/191(32)	All: 49/112(44)	All: 35/72(48),<br>SV-PACNS:13/18(72),<br>LV-PACNS: 17/50(34)	All: 16/47(34),<br>SV-PACNS: 8/29(27.6),<br>LV-PACNS: 8/18(44.4)
Treatment Response Differences	CYC ± steroids reduced relapse (HR=0.44/0.47)	Not directly reported	Mycophenolate mofetil showed better outcomes than CYC + PDN (Rankin 4–6: 8% vs. 37%);Oral and IV CYC equally effective (response rate: 85% vs. 73%)	Maintenance therapy associated with prolonged remission (OR=4.32, p=0.002) and better functional status (OR=8.09, p<0.0001);Gadolinium	Treatment regimen did not predict outcomes/relapses;Delay to treatment associated with higher relapse (p=0.018)	Treatment delay was independent risk factor for poor 1-year prognosis (SV-PACNS: OR=1.012, p=0.02; LV-PACNS: OR=1.048, p=0.04);SV-PACNS showed "high

Comparison Dimension	German Cohort (n=163)[1]	Cleveland Clinic Cohort, USA (n=34)[2]	Mayo Clinic Cohort, USA (n=191)[3]	French Cohort (n=112)[4]	Indian Cohort (n=72)[5]	Henan Provincial People's Hospital Cohort, China (n=47)
				enhancements negatively associated with remission (OR=0.20, p=0.0007)		baseline injury but better recovery"
Mortality Rate (%)	All: 29/163(17.8);<br>Biopsy-confirmed：17/105(16）;<br>Angiogram-confirmed:12/58(21）	-	All: 54/191(28)	All: 9/112(8)	All: 8/72(11.1),<br>SV-PACNS: 3/18(17),<br>LV-PACNS: 7/50(14)	All:2/47(4.3),<br>SV-PACNS: 1/29(3.4),<br>LV-PACNS: 1/18(5.6)
V. Subtype-Specific Differences						
Subtype-Specific Differences	Biopsy-confirmed: More seizures/microhemorrhages/mass lesions, less MRA positivity;<br>Angiogram-confirmed: More hypertension/smoking/hemiparesis/infarctions/MRA positivity.	SVV: More meningeal/parenchymal enhancement, tumor-like lesions,biopsy positivity ;<br>LMVV: More brain infarcts, strong/concentric VWE, DSA use.	Biopsy-confirmed: More cognitive dysfunction, gadolinium-enhanced lesions , abnormal CSF ;<br>Angiogram-confirmed: More infarct, stroke history.	SVV: More relapses, gadolinium enhancements;<br>LMVV: More infarcts, DSA abnormalities.	SV-PACNS: Younger age, longer diagnosis delay, higher relapse, worse long-term outcome;<br>LV-PACNS: More stroke, abnormal HR-VWI.	SV: Mainly characterized by intracerebral hemorrhage, mass-like lesions, and a high positive rate of brain biopsy;LV: Mainly characterized by infarcts, numbness or weakness of limbs, vascular stenosis, and circumferential vessel wall enhancement.

Note: Key abbreviations: SVV = small vessel vasculitis, LMVV = large/middle vessel vasculitis, HR-VWI = high-resolution vessel wall imaging, DSA = digital subtraction angiography , GC =

glucocorticoid, CYC = cyclophosphamide, IV = intravenous

References

1. Fisse, A.L., et al. (2025) *Disease Characteristics and Treatments Associated with Outcome in Primary Angiitis of the Central Nervous System-A Multicenter Cohort Study in 163 Patients.* Ann Neurol.http://dx.doi.org/10.1002/ana.27295.
2. Shimoyama, T., et al. (2023) *Clinical characteristics, brain magnetic resonance imaging findings and diagnostic approach of the primary central nervous system vasculitis according to angiographic classification.* Clin Exp Rheumatol. **41**(4): 800-811.http://dx.doi.org/10.55563/clinexprheumatol/a9886f.
3. Salvarani, C., et al. (2020) *Long-term remission, relapses and maintenance therapy in adult primary central nervous system vasculitis: A single-center 35-year experience.* Autoimmun Rev. **19**(4): 102497.http://dx.doi.org/10.1016/j.autrev.2020.102497.
4. de Boysson, H., et al. (2018) *Treatment and Long-Term Outcomes of Primary Central Nervous System Vasculitis.* Stroke. **49**(8): 1946-1952.http://dx.doi.org/10.1161/strokeaha.118.021878.
5. Paramasivan, N.K., et al. (2024) *Primary Angiitis of the CNS: Differences in the Profile Between Subtypes and Outcomes From an Indian Cohort.* Neurol Neuroimmunol Neuroinflamm. **11**(4): e200262.http://dx.doi.org/10.1212/nxi.0000000000200262.
